# Responses of Intrinsic Water-use Efficiency and Tree Growth to Climate Change in Semi-Arid Areas of North China

**DOI:** 10.1038/s41598-017-18694-z

**Published:** 2018-01-10

**Authors:** L. U. Weiwei, Y. U. Xinxiao, J. I. A. Guodong, L. I. Hanzhi, L. I. U. Ziqiang

**Affiliations:** Beijing Forestry University, Key Laboratory of State Forestry Administration on Soil and Water Conservation, Beijing Engineering Research Center of Soil and Water Conservation, Beijing, 100083 China

## Abstract

Tree-level intrinsic water-use efficiency (iWUE) is derived from the tree-ring ^13^C isotope composition (δ^13^C) and is an important indicator of the adaptability for trees to climate change. However, there is still uncertainty regarding the relationship between long-term forest ecosystem carbon sequestration capacity and iWUE. To determine whether elevated atmospheric CO_2_ concentration (Ca) increase iWUE and tree growth (basal area increment, BAI), dendrochronological methods and stable isotope analyses were used to examine annual changes in the tree-ring width and carbon isotope composition (δ^13^C) of *Platycladus orientalis* in northern China. The iWUE derived from δ^13^C has increased significantly (p < 0.01). Long-term iWUE trend was largely and positively driven by the elevated atmospheric CO_2_ concentration and temperature. We observed a general increase in averaged BAI, which had significant positive correlation with iWUE (R^2^ = 0.3186, p < 0.01). Increases in iWUE indeed translated into enhanced *P*. *orientalis* growth in semi-arid areas of northern China. Elevated atmospheric CO_2_ concentration significantly (p < 0.01) stimulated *P*. *orientalis* biomass accumulation when Ca was less than approximately 320 ppm in the early phase; however, this effect was not pronounced when Ca exceeded 320 ppm.

## Introduction

Human activities, including the burning of fossil fuels and changes in land use, have altered the composition of the atmosphere and influenced terrestrial biomes via climate change, particularly in developing countries^1,2^. During the last 150 years, the CO_2_ mole fraction in the atmosphere has increased from 280 to 380 µmol mol^−1,^ ^3^. Long-term forest productivity and carbon sequestration capacity are affected by climate change, and have become a global concern^4^. The ability to use water and nutrient resources plays a significant role in the adaptation of taxa in terrestrial biomes to variations in the environment^5^. Tree-level intrinsic water-use efficiency (iWUE) is estimated as the ratio of net photosynthetic CO_2_ assimilation (A) to stomatal conductance (g_s_). iWUE is an important component of water-carbon coupling and process management in terrestrial ecosystems^6^, as well as a way for trees to adapt the changing environment^7,8^. Stomatal conductance is expected to decline as the concentration of CO_2_ in the environment increases^9,10^, which affects the gradient between atmospheric CO_2_ (Ca) and internal leaf CO_2_ (Ci), and in turn determines the ^13^C isotope composition (δ^13^C) of assimilated carbon^5^. The “Suess effect” refers to a change in the ratio of the atmospheric concentrations of heavy isotopes of carbon (^13^C), resulting from the admixture of large amounts of fossil-fuel derived CO_2_, which is depleted in ^13^CO_2_^11^. During carbon fixation, fractionation associated with physical and enzymatic processes causes plant matter to become ^13^C-depleted in comparison with the air. To distinguish variations in the δ^13^C of source CO_2_ from the effects of plant metabolic processes, the δ^13^C signatures of plant organic material are usually translated to carbon isotope discrimination (Δ^13^C), and Δ^13^C is related to the ratio of Ci to Ca during photosynthesis in C3 plants^12^. Thus, any variability in the iWUE is recorded in the carbon isotopes of tree tissues. Because carbon builds up in tissues over time, the δ^13^C values do not represent the iWUE in a given instant but rather the average iWUE during the formation of tree-tissue organic matter.

Many studies have shown that rising atmospheric CO_2_ concentrations can speed up tree growth by stimulating photosynthesis in a process known as “CO_2_ fertilization”; however most of these studies were conducted over short time periods in growth chambers with seedlings or young plants^7,13,14^. The long-term responses of forests to gradually changing environment are complicated by factors that affect several fluxes at the same time, and by complex interactions among the factors^15,16^. For example, the direct response of photosynthesis to an increase in atmospheric CO_2_ concentration will require additional N and other nutrients, and the production response will therefore decline as nutrient limitation progresses. Results derived from short-term experiments may not represent longer-term responses of plants to multiple factors in the natural environment^4^. Compared with short-term experiments, tree ring information provides a more reliable summary of physiological responses to environmental variations over time and can be used to predict the behavior of mature trees^17–21^. Studies of tree-ring stable carbon isotopes at a global scale have found variable tree responses to climate change, reflecting the complex relationship between environmental variability and tree growth, and suggest that adaptation strategies vary widely across species and sites^22–25^. Our knowledge of the long-term relationship between iWUE and tree growth remains incomplete. Tree rings are ideal proxies for assessing changes in physiology and growth over time and under different environmental conditions.

The Yanshan Mountains of northern China, between the Mongolia Plateau and the North China Plain, are forested and dominated by *P*. *orientalis*. This area plays a significant role in the global carbon cycle due to its typical monsoon climate. *Platycladus orientalis*, an evergreen coniferous tree species that is endemic to China, is the main tree species used for afforestation in the semi-arid areas of northern China. Climate change research in China over the last century has focused on the importance of the adaptation strategies and productivity of typical evergreen conifers in the semi-arid region of northern China^13^. In this study, we examined annual changes in carbon isotope composition and tree-ring width, investigating trends in tree-ring carbon discrimination (Δ^13^C) and iWUE over the last century in *P*. *orientalis* of the Yanshan Mountains. The specific goals of the study were to (1) determine whether tree-level iWUE increased over time (i.e., across subsequent calendar years) and whether that translated into tree growth and (2) assess how iWUE and tree growth (as measured by the basal area increment [BAI]) responded to changes in atmospheric CO_2_ concentrations, annual temperature, and precipitation.

## Materials and Methods

### Study sites, tree sampling, and analysis

The study was carried out in the Hongmenchuan watershed (40°26′N, 117°10′E) of northern China, which is an important ecological refuge for Beijing and more than two million people. The Hongmenchuan watershed, located in the transition zone of the North China Plain and the Mongolia Plateau, has a complex geology and belongs to the Yanshan Mountain range (200–1200 m a.s.l.). The region is characterized by a sub-humid, warm and temperate continental monsoon climate. The vegetation in the Hongmenchuan watershed is natural forests with no forest management, dominated by *P*. *orientalis* with a density of 739 trees per ha, along with the co-dominant tree species *Pinus tabulaeformis*, with a density of 116 trees per ha; these data were obtained through sample plot investigation (data not shown).

The detailed information for each plot is shown in Table 1 ^26^. *P*. *orientalis* is mainly distributed in areas with an altitude of 200 m–600 m, on shady and sunny slopes^27^. Therefore, we selected 10 typical plots with altitudes ranging from 332 m to 562 m located on each slope aspect. Within each plot, four *P*. *orientalis* trees that were at least 90 years of age were selected for sampling. Two or three cores were taken from each tree at breast height (1.3 m) from different sections of the stem using a 5-mm increment borer (a total of 92 cores were collected). To prevent contamination from other carbon sources, the collected samples were stored in glass tubes.

**Table 1:** Geographical, topographical, and structural characteristics of the sampled sites. The numbers 1 to 10 represent the site codes. DBH (cm) is diameter at breast height. Values shown are the means ± SE.

Site code	Latitude (N)	Longitude (E)	Aspect	Elevation (m)	Slope (°)	DBH (cm)	Height (m)
1	40°28′	117°8′	NE	562	30	21.4 ± 3.7	18.5 ± 1.5
2	40°28′	117°7′	NE	537	16	20.9 ± 4.3	18 ± 1
3	40°27′	117°9′	NW	496	19	22.3 ± 4.6	20.5 ± 1.5
4	40°26′	117°10′	N	435	26	20.7 ± 2.9	17 ± 2
5	40°25′	117°8′	SE	508	27	21.6 ± 4.4	19.5 ± 1
6	40°26′	117°7′	SW	522	23	22.5 ± 3.4	20.5 ± 1
7	40°25′	117°7′	SE	369	25	23.4 ± 3	22.5 ± 1.5
8	40°24′	117°5′	SW	332	21	22.3 ± 4.5	21.5 ± 2
9	40°24′	117°6′	S	414	22	23.9 ± 3.7	22 ± 1.5
10	40°25′	117°4′	SE	420	20	21.2 ± 2.8	20.5 ± 1

### Ring-width, BAI, and stable carbon isotope analysis

The core samples were sanded with grain paper that varied from 100 to 800 mesh to make the tree-ring more clearly visible for cross-dating. Then, COFECHA was used to check dating, find errors in the individual series, and improve the cross-dating accuracy^7,28^. Eight of the cores had rotted or had been damaged by woodworms, and 11 of the cores were missing rings, which would cause inaccuracies in the test data. Cores with poor quality were excluded from analysis. Thus, only 71 cores were selected for analysis, and these were divided into two sections: 35 cores were used for width measurements, and the remaining 36 cores were used to evaluate stable carbon isotopes. After natural drying, fixation, and grinding, tree ring widths were measured at a resolution of 0.01 mm from each of the cores using LINTAB 6 measurement equipment (Frank Rinn, Heidelberg, Germany) and the data were analyzed using Time Series Analysis and Presentation (TSAP) software package (Frank Rinn, Heidelberg, Germany). Cross-dating of the tree-ring data was verified using COFECHA, which assesses the quality of cross-dating and the measurement accuracy of tree-ring series using a segmented time-series correlation technique^28^.

Tree-ring width decreases with age in mature trees due to the diameter growth of the stem. Therefore, BAI is more appropriate than DBHI (diameter at breast height increment) for modeling tree growth and forest productivity. BAI was estimated according to the following formula:

1 $${\rm{BAI}}={\rm{\pi }}\times ({R}_{n}^{2}-{R}_{n-1}^{2})$$

where *R* is the radius at breast height and *n* is the year of tree-ring formation. We measured tree-ring width of 35 cores included 12 tree-ring cores more than 150 years older. We calculated BAI with the beginning width in 1855 calendar year, then we gave up the first 25 years data of each core considering the less number of sample cores and “juvenile age effects” might rise the errors of tree-ring widths (similar consideration applied to the analysis of tree-ring δ^13^C)^13^. Then we calculated the BAI between 1880 to 2014 of each tree.

Prior to the isotope analysis, the first 25 years of each core were removed to avoid “juvenile age effects” on the tree-ring isotopes^13^. In this study, pooling was necessary because *P*. *orientalis* is a slow-growing conifer with very narrow rings, making it difficult to obtain enough material to measure the individual isotopic series. In addition, the small ring size would have created an unacceptably high risk of measurement error. Several recent studies have tested and proved the representativeness of pooled isotopic series for tree-ring δ^13^C compared to individual isotopic series^29–34^. The cores were divided into individual rings using a scalpel to cut along ring lines under a stereomicroscope (40× magnification) and rings within the same year were pooled. Earlywood and latewood were not separated for the isotopic analyses, as recent studies have shown that there is no difference between the two wood types at an isotopic level^35,36^. Each pooled sample was ground into a powder using a ball mill, followed by sieving through an 80-mesh sieve. Cellulose was extracted from all samples to avoid isotope variations during the iWUE calculation, so that they were based purely on the changes in the relative abundance of individual wood constituents that typically have different isotope signatures^37^. The method for cellulose extraction was modified from Loader^38^. Cellulose samples and whole wood samples were used for δ^13^C analysis using a high temperature reactor (HT-O, HEKAtech GMBH, Wegberg, Germany) coupled to a DELTAplus XP Mass Spectrometer (ThermoFinnigan, Thermo Fisher Scientific Inc., Waltham, MA, USA). Each sample was analyzed three times, and the average values were calculated.

The results from the isotope ratio deviations are presented using the common δ notation:

2 $$\delta =(\frac{{R}_{sa}}{{R}_{re}}-1)\times 1000\textperthousand $$

where R refers to the ratio of the ^13^C to ^12^C isotopes in the sample (‘sa’) and the reference [‘re’, compared to the PDB (Pee Dee Belemnite) standard].

Therefore, cellulose Δ^13^C was calculated as follows:

3 $${{\rm{\Delta }}}^{13}{\rm{C}}=\frac{{{\rm{\delta }}}^{13}{{\rm{C}}}_{a}-{{\rm{\delta }}}^{13}{{\rm{C}}}_{p}}{1+{{\rm{\delta }}}^{13}{{\rm{C}}}_{p}}$$

where δ^13^C_a_ and δ^13^C_p_ are the ratios in atmospheric CO_2_ and tree-ring cellulose, respectively.

The relative rates of carbon fixation and stomatal conductance are the primary factors that determine Δ. According to the model proposed by Farquhar^12^, Δ^13^C and δ^13^C have different trends.

4 $${{\rm{\Delta }}}^{13}{\rm{C}}={\rm{a}}+({\rm{b}}-{\rm{a}})\frac{{C}_{i}}{{C}_{a}}$$

where *a* is the discrimination against ^13^CO_2_ during CO_2_ diffusion through the stomata (*a* = 4·4‰), *b* is the discrimination associated with carboxylation (*b* = 27‰), and *C*i and *C*a are the intercellular and ambient CO_2_ concentrations, respectively.

Fick’s law is as follows:

5 $${\rm{A}}={g}_{C{O}_{2}}({C}_{a}-{C}_{i})$$

The ratio of leaf conductance to water vapor is 1.6 *g* CO_2_, and the change in ^13^C can be related to the ${A/g}_{{{\rm{H}}}_{2}{\rm{O}}}$ ratio as follows:

6 $${{\rm{\Delta }}}^{13}{\rm{C}}={\rm{a}}+({\rm{b}}-{\rm{a}})(1-1.6\frac{A}{{C}_{a}{g}_{{H}_{2}o}})$$

iWUE was calculated using the following equation:

7 $${\rm{iWUE}}={C}_{a}\frac{b-{{\rm{\Delta }}}^{13}{\rm{C}}}{1.6(b-a)}$$

### Climate data, CO_2_ records, and δ^13^C_a_

**Figure 1:**
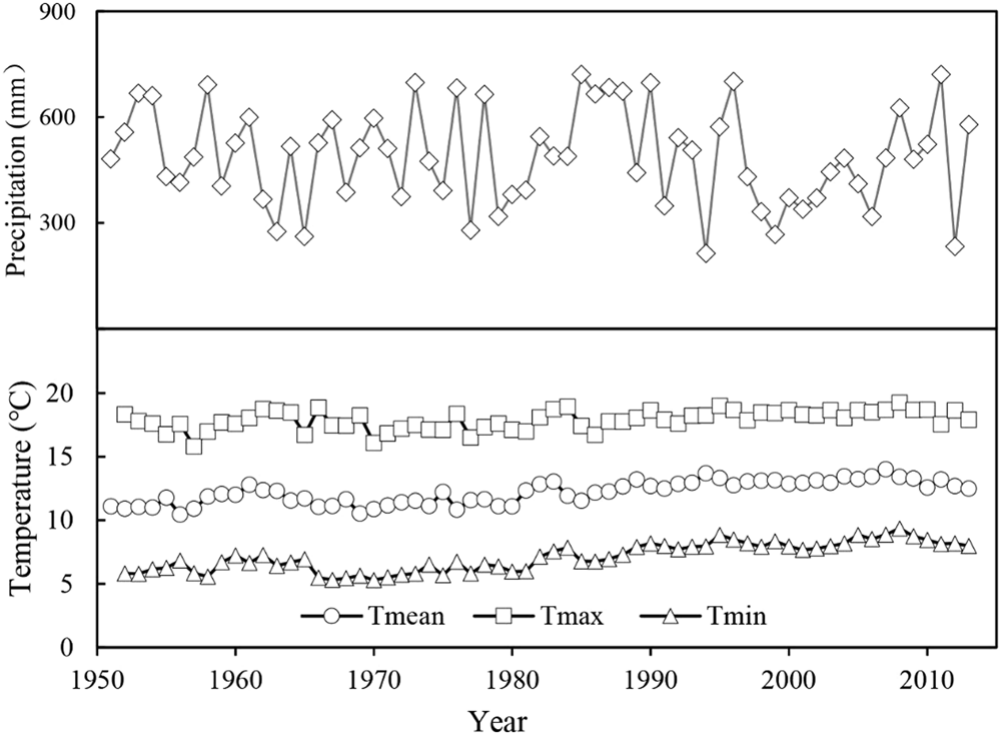
Annual observations of meteorological conditions during the study period: precipitation and mean (T_mean_), minimum (T_min_), and maximum (T_max_) temperature between 1951 and 2014.

All weather data were obtained from the Miyun National Forest Farm Meteorological Station (117°07′E, 40°22′N) located at 352 m a.s.l., 5 km from the study area. The data were checked for quality and consistency, and included the annual minimum, mean, and maximum temperatures, as well as annual precipitation, from 1951 to 2014. As Fig. 1 shows, mean annual temperatures in the study area ranged from 10.45 °C to 14.02 °C, with an average temperature of 12.2 °C, and there was an increasing trend of 0.039 °C per year during the study period. A similar trend was seen for the minimum annual temperatures and no obvious change was observed in maximum temperature. Annual precipitation exhibited a slight decrease over the study period (−3.4625 mm per year), and differed significantly among calendar years, ranging from 261.4 mm to 1404.6 mm. Annual atmospheric CO_2_ concentration records have been monitored at the Shangdianzi Atmospheric Background Station since 1981. Data prior to 1981 were obtained from the National Oceanic and Atmospheric Administration. We used δ^13^C_a_ data that were recorded at the Shangdianzi National Atmospheric Background Station (117°07′01″E, 40°39′00″N), located at 112 m.a.s.l., 31.8 km from the study area.

### Statistical analysis

To model tree growth, we measured all cores ring widths and calculated BAI of each tree and then averaged them. One-way Analysis of Variance (ANOVA) was used to examine the BAI variation among trees. We used pooled wood samples, mixing material of the same cross-dated ring for measuring annual δ^13^C and then calculated iWUE. Regression analyses and t-test were used to identify significant trends in δ^13^C, Δ^13^C, iWUE, and BAI from 1880 to 2014, and to test the relationships between iWUE and BAI, between iWUE and environmental factors (include atmospheric CO_2_ concentration, annual temperature and precipitation). R^2^ and slope *p*-values were also estimated. All statistical analyses were carried out using the SPSS 11.0 statistical software package (SPSS, Chicago, IL, USA).

## Results

### Patterns of δ^13^C, Δ^13^C, iWUE, and BAI

**Figure 2:**
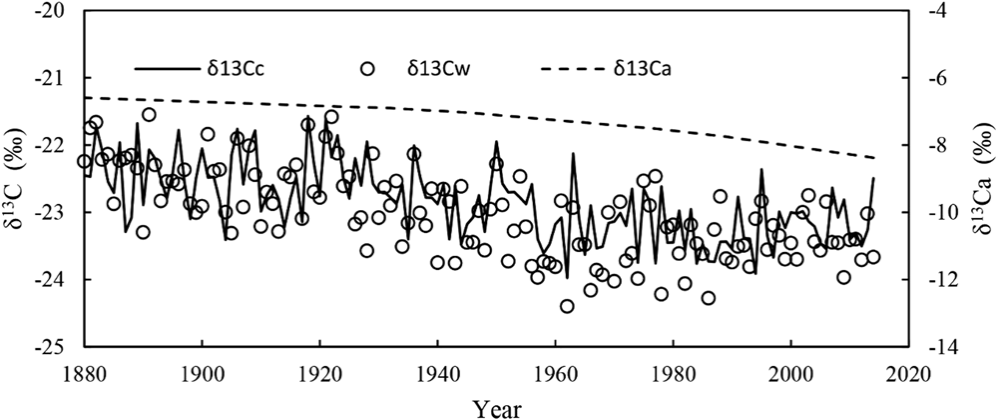
Annual variations in: the tree ring cellulose stable carbon isotope (δ^13^Cc, polyline) record for *P*. *orientalis*, the tree ring whole wood stable carbon isotope (δ^13^C_w_, circles) record for *P*. *orientalis*, and the atmospheric stable carbon isotope (δ^13^C_a_, dotted line) between 1880 and 2014. We connected the annual δ^13^Cc points to a polyline, for convenient comparison with δ^13^C_w_.

Figure 2 shows the whole wood δ^13^C_w_ and cellulose δ^13^C_c_ trends under decreased stable atmospheric carbon isotopes (δ^13^C_a_) (“Suess effect”) from 1880 to 2014. The t-test revealed that a significant difference between δ^13^C_w_ and δ^13^C_c_, implying that δ^13^C_c_ cannot be replaced by δ^13^C_w_. We detected a decreased trend in both δ^13^C_w_ and δ^13^Cc curves. The δ^13^C_c_ pattern could be divided into approximately three periods, i.e., two plateau periods from 1880 to 1917 and from 1975 to 2014 and one decreasing period from 1918 to 1974. The δ^13^C_c_ declined from 1918 to 1974 and exhibited considerable fluctuation during that time, and both the maximum (−21.57‰, 1918) and minimum (−23.97‰, 1962) values were detected in that period. By comparison, there was no clear trend in the other two periods, during which time the δ^13^C_c_ curve was relatively stable.

**Figure 3:**
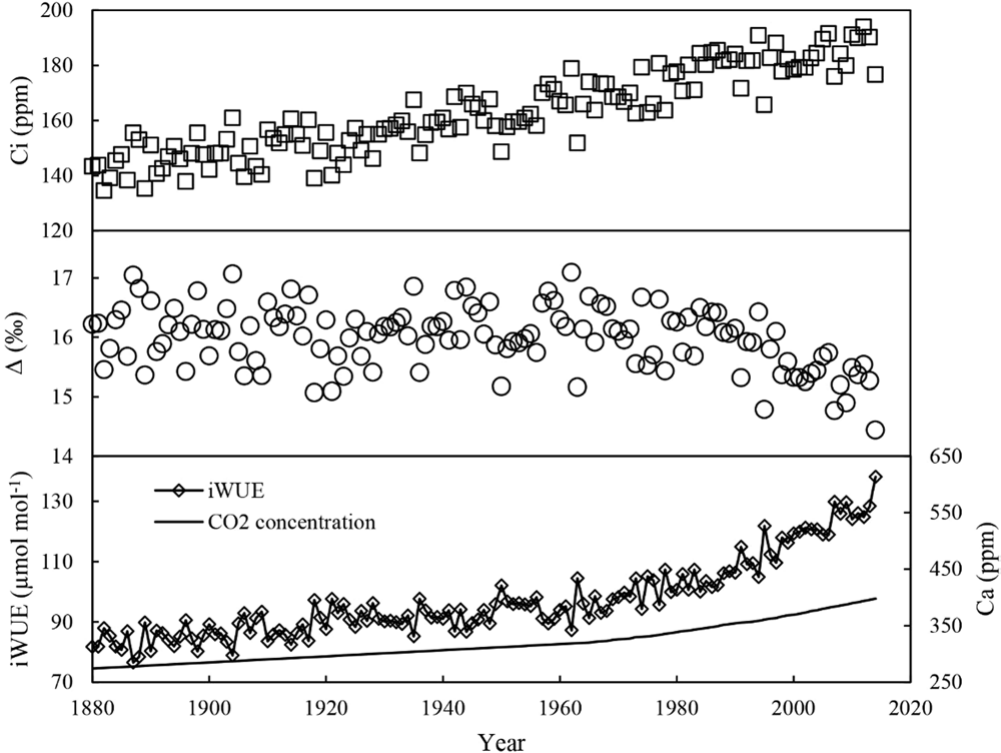
Annual variations in the tree ring indices for *P*. *orientalis*: (**a**) intercellular CO_2_ concentrations (Ci), (**b**) tree ring cellulose carbon isotope discrimination (Δ^13^C), (**c**) intrinsic water use efficiency (iWUE) derived from tree ring cellulose carbon isotopes (δ^13^Cc) and atmospheric CO_2_ concentrations between 1880 and 2014.

iWUE increased significantly, particularly since 1974, with changes in the Ci and CO_2_ concentrations (Fig. 3), while the discrimination (Δ^13^C) trends exhibited the opposite pattern (a decrease). There was a significant increase in iWUE, at a rate of 0.49 μmol mol^−1^ per year, throughout the last century (p < 0.05). iWUE ranged from 82.35 μmol mol^−1^ (1920) to 138.12 μmol mol^−1^ (2014) during the study period, and a significant increase was detected from 1974 to 2014, at a rate of 1.1 μmol mol^−1^ per year. Higher Ci values were associated with lower discrimination (Δ^13^C) and higher iWUE.

**Figure 4:**
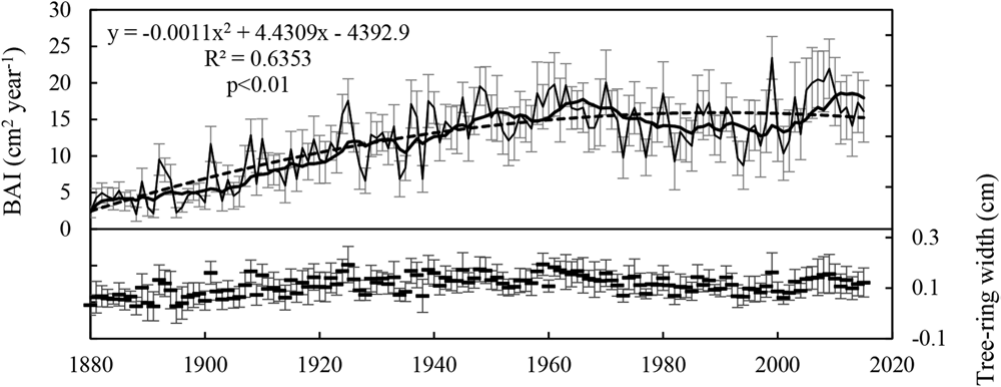
Annual variations in tree-ring width and averaged basal area increment (BAI, cm^2^) for *P*. *orientalis*. The solid line represent the averaged BAI and the bold curve represents the smoothed results using a 10-year fast Fourier transform (FFT) filter to emphasize long-term variations. The dotted line is the trend line. Error bars indicate the standard error of the means.

Tree-ring width tended to fluctuate during the last century, ranging from 0.029 cm to 0.193 cm, with an average of 0.1 cm (Fig. 4). Tree-ring width increased initially (from 1880 to 1961) and then decreased from 1962 to 2001, as shown in Fig. 4. Since 2002, there has been a slight increase in tree-ring width. The averaged BAI showed a significant quadratic curve increase from 1880 to 2014 (R^2^ = 0.6353, p < 0.01), especially during the early years of growth, after which the increasing trend weakened since 1960. The increase in BAI in the earlier decades ranged from 2.01 cm^2^ to 19.7 cm^2^, which accounted for approximately 82.7% of the total increase, indicating that the trees grew rapidly during that period. The rapid increasing phase was followed by a decrease in BAI, which has fluctuated since the 1960s.

### Relationships between iWUE and temperature, precipitation, and atmospheric CO_2_

**Figure 5:**
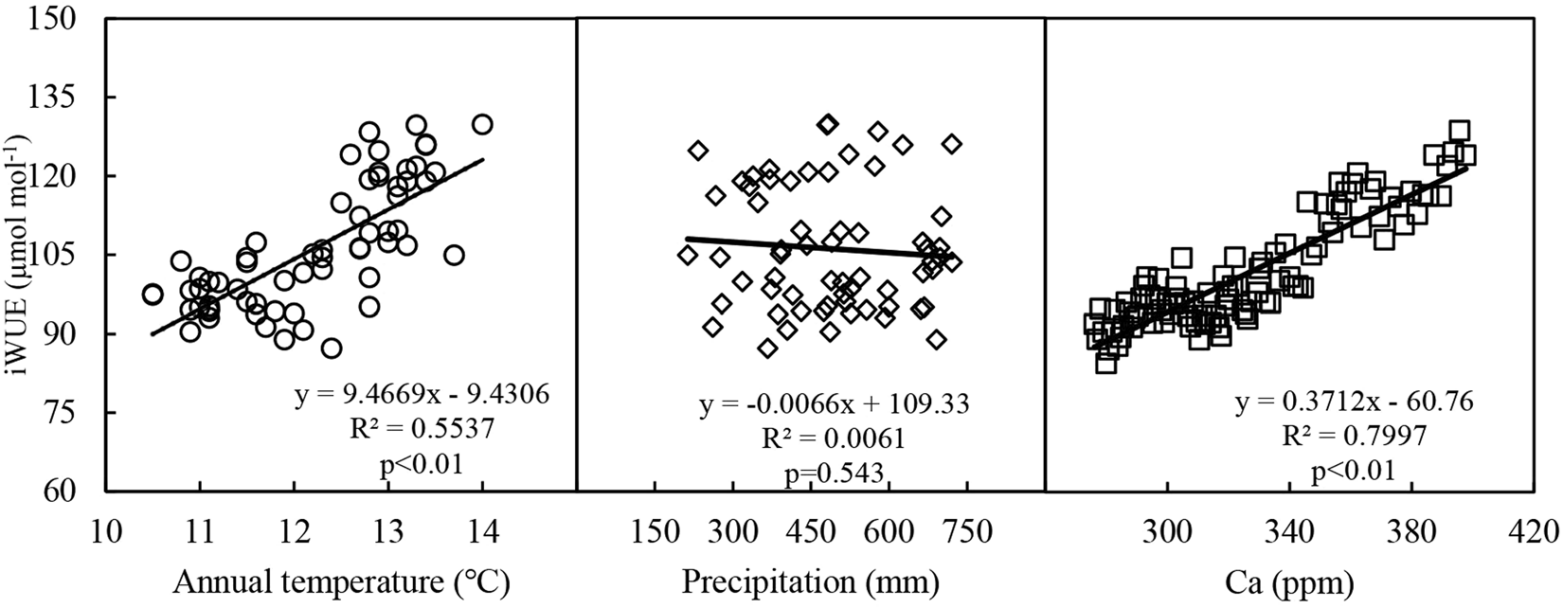
Relationships between iWUE and annual temperature, precipitation, and atmospheric CO_2_ concentration (Ca). Lines indicate the change trends. The regression equations (iWUE and annual temperature, iWUE and annual precipitation, iWUE and annual atmospheric CO_2_ concentration), coefficients, and levels of significance are also shown.

Correlations between iWUE and annual temperature were significant and positive (R^2^ = 0.5537, p < 0.01, Fig. 5). The slope of the regression was 9.4669, indicating that every 0.1 °C increase in annual temperature was accompanied by an by a 1.21 μmol mol^−1^ increase in iWUE per year. Annual precipitation fluctuated greatly, ranging from 250 mm to 800 mm, and decreased in recent years. However, no significant relationship between iWUE and precipitation was observed. The results of the correlation analysis indicated that iWUE was positively correlated with elevated Ca (*r* ^2^ = 0.7997, p < 0.01).

### Relationships between BAI and temperature, precipitation, Ca, and iWUE

**Figure 6:**
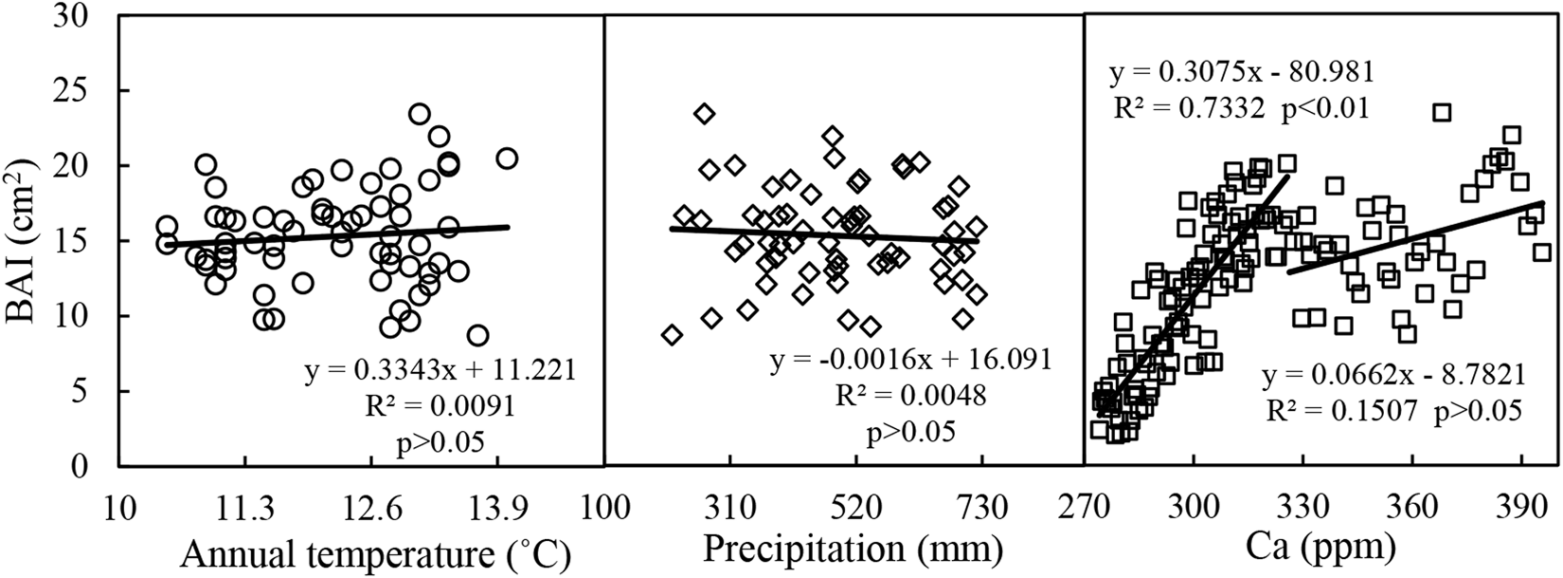
Relationships between averaged BAI and annual temperature (1951–2014), precipitation (1951–2014), and atmospheric CO_2_ concentration (Ca) (1880–2014). Lines represent the change trends. Regression equations (averaged BAI and annual temperature, annual atmospheric CO_2_ concentration), coefficients, and levels of significance are also shown.

**Figure 7:**
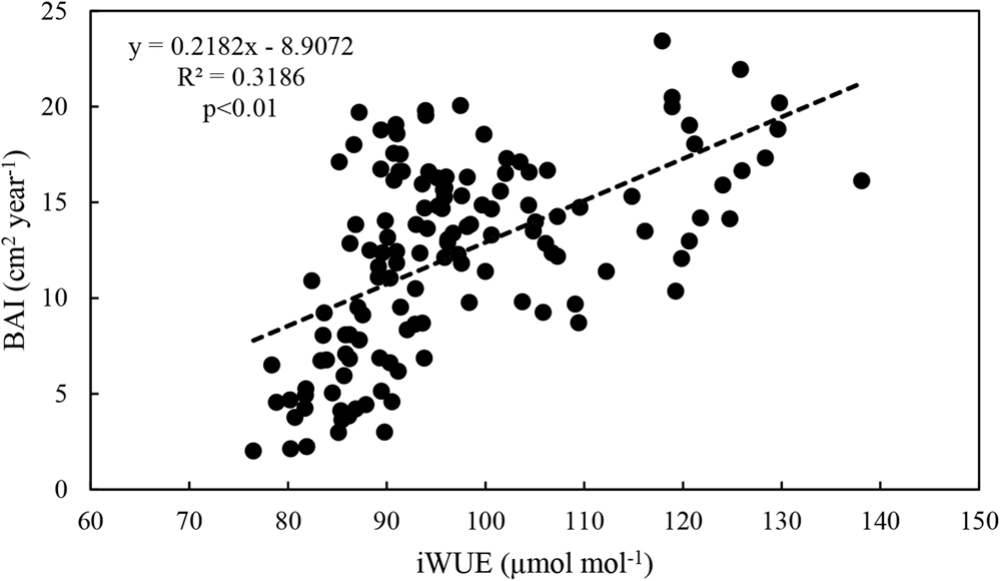
Relationship between averaged BAI and iWUE. Lines represent the change trends. Regression equations (averaged BAI and iWUE), coefficients, and levels of significance are also shown.

No significant relationship was observed between BAI and annual temperature (R^2^ = 0.0091, p > 0.05, Fig. 6), indicating that the influence of annual temperature on BAI was not as strong as that on iWUE. In addition, the relationship between BAI and precipitation were weak and not significant (R^2^ = 0.0048, p > 0.05). There was a nonlinear relationship between BAI and Ca. Elevated atmospheric CO_2_ concentration significantly (p < 0.01) stimulated *P*. *orientalis* biomass accumulation when Ca was less than approximately 320 ppm in the early phase; however, this effect was not pronounced when Ca exceeded 320 ppm. The correlation between BAI and iWUE was positive and significant (p < 0.01, Fig. 7).

## Discussion

We found that in the first half of the last century, the error in the *P*. *orientalis* tree-ring δ^13^C was relatively high, but it decreased gradually over time. It seems that saplings exhibited varying responses to environmental change; this, can be described as the “juvenile effect”. One reason for this age-related trend in δ^13^C could be the recycling of respired air that has already been depleted of ^13^C by young trees growing close to the forest floor. Plant tissue δ^13^C is significantly lower than that found in the atmosphere because atmospheric CO_2_ contains 98.9% of light-stable carbon isotope and 1.1% heavy-stable carbon isotope, and the light-stable carbon isotope is preferentially absorbed during photosynthesis, resulting in carbon isotope fractionation, which is known as the “carbon isotope effect”^39^. This process may be influenced by biotic factors, such as tree age and height, as well as abiotic factors such as meteorological conditions (radiation regime, temperature, precipitation, atmospheric pressure, etc.), CO_2_ concentration, site conditions (water availability, nutritional constraints, elevation and slope exposure, etc.), and forest management and anthropogenic perturbations (fires, climate change, etc.)^31,39–41^. Chen *et al*.^42^ suggested that altitudinal variation in δ^13^C may be negative or unchanged for plants growing in arid and/or semiarid regions, and atmospheric pressure has a negative effect on plant δ^13^C. Di Matteo *et al*.^43^ conducted a replicated field trial to characterize the interacting effects of aspect and intensifying drought on foliar δ^13^C and to quantify temporal variation in stomatal conductance. They found a significant reduction in stomatal conductance (p < 0.01) as the drought increased along a sunny slope, and foliar δ^13^C increased significantly (p < 0.01). In this study, we selected 10 typical plots, for which the altitude ranged from 332 m to 562 m and located on two slope aspects, to carry out our study with the original intention of understanding the average conditions of iWUE and tree growth of *P*. *orientalis* in northern China. Therefore, we did not take into account the effect of differences in exposure and elevation on tree-ring δ^13^C.

During the study period, the *P*. *orientalis* iWUE was significantly and positively correlated with annual temperature. This result agrees with research conducted on other tree species around the world^7,37,44^. This phenomenon can be explained by plant ecophysiology. First, when plants are subjected to high temperatures their stomata partially close, and then g_s_ decreases to prevent an excessive loss of water, thereby reducing the internal CO_2_ concentration of the leaves. The absorption and utilization rate of internal leaf CO_2_ increase to maintain high photosynthetic rate (An). At the same time, an increase in temperature causes an increase in the vapor-pressure deficit and in the transpiration rate (Tr); accordingly, iWUE and temperature were positively related in this study. The number of days for which the daily maximum temperature was higher than 30 °C increased substantially in the study area each year, together with the frequency of extreme temperature values, leading to reduced stomatal conductance and altering the environmental and physiological functions of *P*. *orientalis*.

Following the second industrial revolution, atmospheric CO_2_ concentrations have gradually increased, rising from 276.1 ppm to 397.6 ppm in northern China according to the China Global Atmosphere Watch Baseline Observatory. We found that the iWUE increased significantly under elevated atmospheric CO_2_ concentrations. Our findings are consistent with other studies conducted on the effects of elevated CO_2_ on leaf physiological activity, which demonstrating that increased CO_2_ promotes WUE (determined by traditional measurement methods: weighing, gas exchange, etc.)^45,46^ Jackson *et al*.^47^ found that increased CO_2_ led to 50% decreases in stomatal conductance and transpiration, while the photosynthetic rate increased by 70% and WUE nearly doubled with sufficient illumination. Curtis *et al*.^48^ found that leaf photosynthetic products increased by approximately 50% under elevated CO_2_ concentrations after combining 38 groups of experimental data. Gagen *et al*.^49^ noted that northern boreal *Pinus sylvestris* may have reached a threshold with respect to its ability to increase WUE under rising atmospheric CO_2_ concentrations.

In this study, we used the averaged BAI to characterize trees growth. Then we did the ANOVA between averaged BAI and each tree’s BAI, a total of 35 pairs of data series, found that the 77.1% pairs had no significant difference. Thus the variation among trees could be ignored and averaged BAI could represent most trees growth in this region. The averaged BAI showed a significant increasing tendency (p < 0.01); however, the coefficient of determination between BAI and iWUE was relatively low (R^2^ = 0.3299), and there was still some difference values between before and after the 1970s. The BAI increased significantly before the 1970s and then fluctuated but did not increase significantly. It seems that there is a limitation beyond which the increase of BAI would abate. Some scholars found that when atmospheric CO_2_ concentration exceeds approximately 350 ppm, the BAI decreased with increasing atmospheric CO_2_ concentration^4^. In this study, we found that the tipping point of the concentration for the BAI response curve was approximately 320 ppm. Thus, there is a contrast between the results of short-term experiments and long-term studies. A notable difference is nitrogen availability, which may positively stimulate leaf area, photosynthesis, and plant growth^50^. However, the effect of nitrogen on plants may be less pronounced or may even become negative over time due to altered soil N transformation processes and progressive nutrient limitations in ecosystems approaching and surpassing N saturation^4,51^. It is likely that these effects caused the difference in the results between short-term experiments and long-term studies.

In addition, there was no clear response of the iWUE to changes in precipitation. In the rocky mountainous area of northern China, precipitation mainly causes increases in runoff with some percolation through the soil. Changes in precipitation typically affect plant growth conditions by altering the soil moisture content^52^. There were no significant changes in precipitation during the study period and only a slight decrease was observed after the year 2000. In any case, the inter-annual variability of rainfall is very high, and we did not found any relationship between annual precipitation and iWUE or BAI. It seems that *P*. *orientalis* is well adapted to dry conditions and can cope withthe limitation posed by dry conditions by increasing iWUE. This finding agrees with that of Niinemets *et al*.^53^ for evergreens that exhibited greater responsiveness to increased CO_2_.

Many studies have focused on the relationship between tree growth and water use efficiency, but it is unclear whether increased water use efficiency translates into greater tree growth^54,55^. Sleen^56^ found that while increasing atmospheric CO_2_ concentrations generally result in more efficient water use in the monsoon forests of Thailand, tree growth still tends to decline. This means that changes in other limiting climatic factors exert stronger opposing effects on tree growth. Wang *et al*.^57^ found that since 1800, tree radial growth was not always associated with increased iWUE in the Xinglong Mountains of northwestern China; in fact, tree growth rates declined during periods of drought and particularly in the past decade. These results suggested that increases in iWUE are unable to compensate for the negative effects of severe limitations on tree radial growth.

In contrast with other studies, we found evidence for long-term increases in BAI that were associated with increases in iWUE. *P*. *orientalis* growth increased as iWUE increased in the mountainous area of northern China due to drought tolerance and high temperature resistance. In addition, the long-term water use efficiency of the *P*. *orientalis* forest ecosystem increased under elevated CO_2_ concentrations and rising temperatures, suggesting that trees overcame the unfavorable factors and made full use of favorable growth conditions. This is mainly because of the special leaf structure, i.e., photosynthetic limitation by mesophyll diffusion varies strongly with the leaf structure^58^. This limitation caused by internal diffusion conductance is expected to be particularly large in sclerophyllous evergreens such as *P*. *orientalis*, which have a structure that enables them to adapt to water limitations^59^. Furthermore, *P*. *orientalis*, supports numerous leaf age classes, leading to high leaf-area indices as 8–13 m^2^ m^−2^ on a projected leaf area basis^60^. With a more robust leaf structure, typical reductions in stomatal conductance observed in response to elevated CO_2_ are likely to alter photosynthesis reduction, and generate a stronger response of iWUE^53,61^. In the context of climate change, the eco-physiological functions of the *P*. *orientalis* forest ecosystem are not inhibited, and maintains a high potential for carbon sequestration.

## Conclusions

Global climate change has had significant impacts on forest ecosystem function worldwide, and these effects vary across regions. Understanding the responses of iWUE and tree growth to climate change has great importance for predicting how plants may respond to climate variations in the future and its implications for forest management and conservation. In our study, we detected a significantly increasing trend in iWUE curves (p < 0.01) and a significant positive correlation between iWUE and BAI (p < 0.01). Long-term iWUE trend could be largely and positively driven by the elevated CO_2_ concentration and temperature. Elevated atmospheric CO_2_ concentration stimulated *P*. *orientalis* BAI accumulation significantly (p < 0.01) when Ca was below about 320 ppm in the early phase. Then the promotion effect were not pronounced when Ca was above 320 ppm. There was not a decrease in the growth of drought-tolerant conifers in northern China, suggesting that growth of *P*. *orientalis* in semi-arid areas benefited from the elevated atmospheric CO_2_ concentrations. The study outcomes may be used to improve forest management and conservation in semi-arid mountainous areas.
